# Hepatic Dendritic Cells in the Development and Progression of Metabolic Steatohepatitis

**DOI:** 10.3389/fimmu.2021.641240

**Published:** 2021-03-23

**Authors:** Nahum Méndez-Sánchez, Jacqueline Córdova-Gallardo, Beatriz Barranco-Fragoso, Mohammed Eslam

**Affiliations:** ^1^Faculty of Medicine, National Autonomous University of Mexico, Mexico City, Mexico; ^2^Liver Research Unit, Medica Sur Clinic & Foundation, Mexico City, Mexico; ^3^Department of Hepatology, Service of Surgery and Obesity Clinic, General Hospital “Dr. Manuel Gea González”, Mexico City, Mexico; ^4^Department of Gastroenterology, National Medical Center “20 Noviembre”, Mexico City, Mexico; ^5^Storr Liver Centre, Westmead Institute for Medical Research, Westmead Hospital and University of Sydney, Sydney, NSW, Australia

**Keywords:** metabolic steatohepatitis, hepatic dendritic cells, reticuloendothelial system, metabolic associated fatty liver disease, lipotoxicity

## Abstract

Metabolic Associated Fatty liver disease (MAFLD) is a global health problem and represents the most common cause of chronic liver disease in the world. MAFLD spectrum goes from simple steatosis to cirrhosis, in between metabolic steatohepatitis with progressive fibrosis, which pathogenesis is not completely understood. Hence, the role of the immune system has become an important fact in the trigger of inflammatory cascades in metabolic steatohepatitis and in the activation of hepatic stellate cells (HSCs). Among, the more studied immune cells in the pathogenesis of MAFLD are macrophages, T cells, natural killer and dendritic cells. In particular, hepatic dendritic cells had recently attracted a special attention, with a dual role in the pathogenesis of MAFLD. These cells have the capacity to switch from a tolerant state to active state inducing an inflammatory cascade. Furthermore, these cells play a role in the lipid storage within the liver, having, thus providing a crucial nexus between inflammation and lipid metabolism. In this review, we will discuss the current knowledge on the dual role of dendritic cells in lipid accumulation, as wells as in the triggering of hepatic inflammation and hepatocytes cell death in metabolic steatohepatitis.

## Introduction

Metabolic associated fatty liver disease (MAFLD), previously known as non-alcoholic fatty liver disease (NAFLD). The paradigm shift to MAFLD is to eliminate the “negative” nomenclature and reflect more appropriately the underlying pathogenesis and enable for the consideration of the coexistence of other chronic liver diseases, including alcoholic liver disease (1–9). MAFLD affects 20–30% of the worldwide population; and is in trajectory to become the leading cause of chronic liver disease, cirrhosis and hepatocellular carcinoma (HCC) globally (10–12). In addition, MAFLD is taking over other liver diseases as the main indication for liver transplantation (13).

The pathogenesis of MAFLD is complex and not yet entirely understood. It is likely shaped by a dynamic interaction between environmental and genetic factors that shape the outcome of the disease (14, 15). Furthermore, the liver is made up of parenchymal liver cells (such as hepatocytes and biliary cells) and non-parenchymal cells [such as liver endothelial sinusoidal cells (LESC), hepatic stellate cells (HSCs), fibroblasts and immune cells (16). A cross-talk and interaction between these cells modulate the MAFLD progression via diverse signaling pathways that activate inflammatory cascades (16–18).

In particular, liver is considered as an important immunological organ, that it is enriched with multiple innate immune cells, including T lymphocytes, natural killer cells (NK), NKT cells, proper macrophages called kupffer cells (KCs), and dendritic cells. Chronic consumption of “western” diet increases circulating levels of multiple toxins and bacterial products by altering gut flora and intestinal permeability in patients and in murine models, with subsequent intra-hepatic activation of hepatic immune cells, which, is a key phenomenon for the initiation of injury. In addition, this overnutrition and high-calorie diet induce insulin resistance, generating a dysfunctional adipose tissue (AT), leading to the breakdown of triglycerides (TGs) and consequently the formation of free fatty acids (FFAs) and glycerol (19, 20). The increase in the circulating FFAs enhances their uptake by the liver leading to lipid accumulation (21). When these FFAs exceed the metabolizing capacity of the hepatic mitochondria, this would cause a mitochondrial dysfunction with consequent oxidative stress; activating inflammatory pathways and causing hepatocytes cell death, along with the secretion of damage associated molecular patterns (DAMPs) perpetuating the damage (17, 18, 21).

Among the hepatic immune cells, the hepatic dendritic cells (HDCs), uniquely, have a migratory capacity, produce particular cytokines and promote the adaptive immune system response and acts as a bridge between the innate and adaptive process (22). HDCs play a central dual role in metabolic steatohepatitis progression and linking metabolism to inflammation. These cells have the capacity to switch from a tolerant state to active state inducing an inflammatory cascade. On the other hand, these cells play a role in the lipid storage within the liver, having, thus providing a crucial nexus between inflammation and lipid metabolism, that are important antigen presenting cells and inductors of inflammatory pathways. In this manuscript, we will review the current knowledge of the role of immune cells in metabolic steatohepatitis, with special focus on hepatic dendritic cells (HDCs).

## Tolerance and Immune Homeostasis Within the Liver

As, the liver is constantly exposed to multiple antigens and bacterial products form the gut (23) it has to preserve a state of an immune homeostasis or tolerance to avoid sustained inflammation and damage. Immune tolerance is maintained by a diversity of hepatic cells such as HDCs, kupffer cells (KC), liver sinusoidal endothelial cells (LSECs) and hepatic stellate cells (HSC) (24). Chronic alcohol consumption or a “western” diet increases circulating levels of bacterial products including lipopolysaccharide (LPS) by altering gut flora and intestinal permeability in patients and in murine models. Subsequent intra-hepatic activation of toll like receptors (TLRs; particularly 2, 4, and 9), a sensor for these products, is a key phenomenon for the initiation of injury. In particular, the liver resident or infiltrating macrophages, the hepatic endothelial cells and the stellate cells express high levels of TLRs and are the primary mediators of TLR-dependent inflammatory responses. Tight regulation of TLRs signaling is essential to avoid unchecked amplification of inflammation (25).

**Figure 1:**
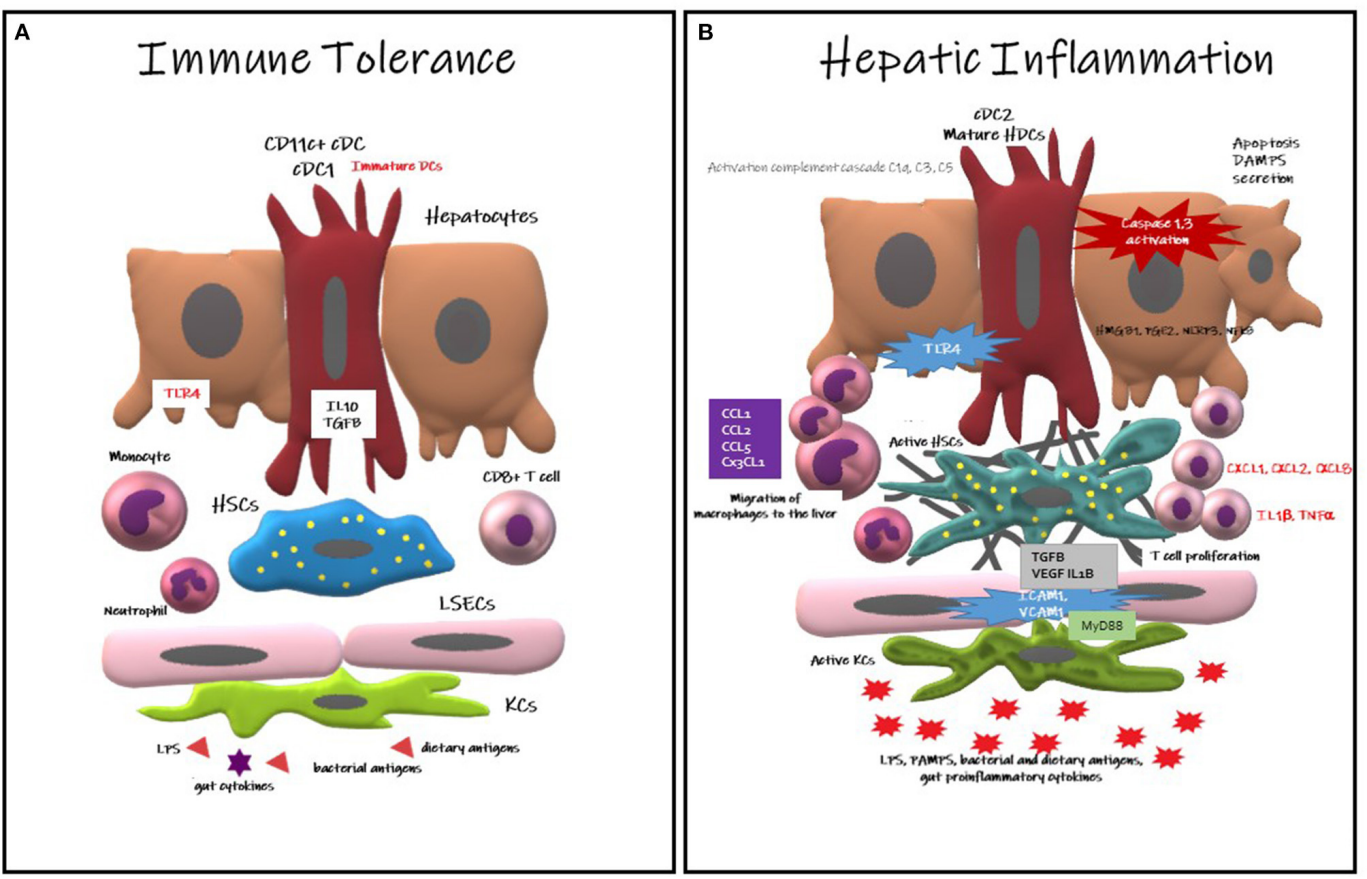
Hepatic Dendritic Cells responses in immune tolerance and in hepatic inflammation. **(A)** Immune Tolerance: CD11c+cDC immature HDCs contribute to immune tolerance secreting IL10, TGFB, limiting T cell expansion and provoking their deletion, reducing the expression of TLR. DCs also contribute to the slot of apoptotic debris. **(B)** Hepatic Inflammation: Mature HDCs (cDC2) provokes a pro-inflammatory environment, recruiting macrophages into the liver. The hepatocytes also contribute to the inflammation secreting HMGB1, PGE2, NRLP3, and activating NFkB pathway. Activation of the inflammasone NLRP3 cause hepatocyte death with the consequent secretion of DAMPS, triggering KCs death. Neutrophils activate Kupffer and endothelial cells upregulating ICAM1 and VCAM1. The complement cascade is also activated contributing to inflammation.

Immune tolerance is mainly induced via a signaling network of cytokines including Interleukin 10 (IL-10), hepatocyte growth factor, retinoic acid and transforming growth factor B (TGFB) (25). IL10 secreted by FoxP3^+^ Treg cells urns the kupffer cells to become more resistance to activation by cytotoxic T Lymphocytes (CTLs) (26). At cellular level, immune homeostasis is mediated by multiple antigen presenting cells. HDCS remain quiescent in the steady state that lead to the suppression of inflammasome activation, a critical pathway in the development of liver damage, and attenuating T-cell activation (26, 27), therefore contribute to maintain a tolerance state within the liver (25). Kupffer cells are also involved in inducing the immune homeostasis state, via their capacity to express programmed cell death ligand 1 (PDL1) inhibiting T-cells activation as well as their ability to secrete IL-10 upon LPS stimulation. In addition, it recruit other regulatory monocytes hindering NFkB, STAT3 and SMAD pathways activations (24) (Figure 1).

## The Reticuloendothelial System Role in MAFLD

The reticuloendothelial system is performed by non-parenchymal cells, which are the main conditioners of acute and chronic liver injury. This group of cells includes HDCs, LSECs and KCs (28).

### Macrophages or Kupffer Cells (KCs)

Hepatic macrophages have dual role in MAFLD, with both pro and anti-inflammatory effects. The two major types of hepatic macrophages are the monocyte-derived macrophages and yolk sac-derived tissue-resident KCs and both are central to hepatic inflammation (29).

The liver macrophages express high levels of TLRs and are the primary macrophages are the first recognition point of gut derived pathogen-associated molecular pattern (PAMPs), reaching via the hepatic portal vein or the local damage-associated molecular patterns (DAMPs) (30). KCs play a role in the early response to injury, while infiltrating macrophages mediate inflammation and fibrosis progression (31). KCs have the capacity to and are the primary mediators of TLR-dependent inflammatory responses. A cross talk between macrophages and other hepatic immune cells, such as natural killer T (NKT) cells contribute to the sustained hepatic inflammation, via inducing the secretion of multiple proinflammatory cytokines, such as IL-4 and IFNγ (24, 25). In addition, activated KCs have been demonstrated to be implicated in the aggravation of hepatic fibrosis in MAFLD (32). The profibrotic functions of resident MPs persist even if the infiltration of monocytes is blocked via MCP-1 inhibition (33). Thus, the combination of both, but neither individually, is sufficient for liver repair.

On the other hand, an anti-inflammatory role for hepatic macrophages is also described. A recent report demonstrated that anti-inflammatory polarized KCs are found to initiate apoptosis of inflammatory KCs by the secretion of IL-10 (34). Mice supplied with probiotics exhibited enhanced anti-inflammatory macrophages (35). These results highlight that the state of polarization of macrophage populations is directly influenced by the microbiota components and exhibits important differences in the balance of inflammatory state in the progression of MAFLD.

### Neutrophils

Neutrophils are recruited via the CXCR1/2 receptors and CXCL1/2 axis (in mice) or IL-8 (in humans), respectively and contribute to progression of hepatic inflammation through multitude of mechanisms (25). Neutrophils secrete myeloperoxidases which is enhanced in metabolic steatohepatitis patients (36) and are toxic to macrophages, thereby contributing to the progression of inflammation and insulin resistance (37, 38). In addition, it can provoke hepatic inflammation via the generation of ROS and proinflammatory cytokines that can further activate KCs and LSECs lead to the upregulation of the expression of adhesion molecules and perpetuation of further neutrophil infiltration (25).

### Natural Killer Cells

Natural Killer T cells (NKT) comprise 10% of liver lymphocytes, which express both NK (CD161 and CD94), and T cell markers and recognize lipid antigens during injury responses for the stimulation of KCs, hepatocytes, and DCs (39). In MAFLD, there is an inverse correlation between NKT cells and intrahepatic lipid (40), although other reports have shown that NKT cells tend to accumulate during MAFLD progression (41). The NKT cells generate both Th1 and Th2 cytokines, so their depletion may result in an increase of Th1 cytokines such TNF-α, IL-2, and IFN-γ (42). In addition, HDCs stimulate the release of proinflammatory cytokines released by NKT cells and become activated after the NKT cells population is eliminated (43).

### Dendritic Cells and Hepatic Dendritic Cells

The DCs are specialized hematopoietic cells derived from monocyte-macrophage DC progenitors originated in the bone marrow and are classified into different subsets based on their developmental, phenotypical, and functional criteria. The DCs are enriched within tissues in contact with the environment, such as the skin, nose mucosa, lungs, stomach, gut, and liver where they express specific surface proteins and exhibit tissue particular functions. They are categorized into classical or conventional DCs (cDCs), plasmacytoid DCs (pDCs), monocyte-derived DCs (MoDCs), and Langerhans cells (LCs) (44). In the liver, HDCs are bone marrow derived cells (22, 45) found preferentially in the periportal and pericentral space. They represent <1% of the non-parenchymal hepatic cells and are a diversified population of hepatic antigen presenting cells (APCs) linked to innate and adaptive immunity and considered as key modulator of hepatic immune system (46). The HDCs express high levels of MHC-Class II molecules (e.g., HLA-DR) and CD45^+^, but are negative for other hematopoietic lineage markers (22).

There are three described subsets in different experimental models of HDCs (CD19–, CD11c+): (1) lymphoid or cDC1 (CD8α+, CD103+, B220–, and CD11b– in mice and CD1c+ in humans), (2) myeloid (CD8α-, B220–, and CD11b+), and (3) plasmacytoid (B220+, CD11b–). The first lymphoid and myeloid are denoted as “conventional” HDCs located at the periportal region and central veins, whereas the plasmacytoid HDCs are located within the liver parenchyma (47). In humans, plasmacytoid DCs are characterized by BDCA-2 and CD123 expression (48), respond to TLR7/8 ligands, and mediate antiviral immunity by secreting type I interferons such as IFN-α but are less potent T-cell inductors. The classical DCs are subdivided in cross presenting CD141 (BDCA-3) DCs (mainly interacting with CD8+ T-cells via MHC-I) and conventional CD1c (BDCA1) DCs (presenting MHC-II bound antigens to CD4+ T-cells) which correspond to human and respectively (22, 25).

The main functions of DCs are to capture antigens, process and present peptides, and migrate to lymphoid organs to induce T-cell mediated immunity (49). Hence, it is considered as one of the most important antigen-presenting cells (APC) to lymphocytes that activate adaptive immune responses (32). Interestingly, HDCs pose bi-phasic roles, in acute damage, they act as protective cells; while in chronic hepatic inflammatory states, they act as perpetuators of the inflammation and the damage (50). HDCs have a pivotal capacity to stimulate inflammatory cascades and cytokine secretion in response to TLR stimulation, as wells as enhancing phagocytosis. During inflammation states, a massive expansion and maturation of HDCs (CD11c+/MHCII^high^/CD103-/CD11b+) occurs via CD80 stimulation (51). On the other hand, these cells can remain quiescent and immature that favors tolerance and avoid triggering aninflammatory cascades in response to damage (46). Interestingly, HDCs determine the balance between tolerance and immunity by their interaction with antigen specific T cells, playing a critical role in regulation of innate and adaptive immunity (52).

#### Dendritic Cells and MAFLD

In metabolic steatohepatitis C57BL/6 mice fed with a methionine/choline-deficient (MCD)-diet there was an overexpression of CD11chigh/F4-80+ DCs pool, but a reduced expression of CD11c+ /MHCII+ /B220+ plasmacytoid DCs (pDCs) and CD11c+ /MHCII+ /CD8a+ lymphocytoid DCs (53). Further, the myeloid HDCs are sub-grouped into mHDC1 (type 1, DC103+/CD11b-) and mHDC2 (type 2, DC 103-/CD11b+) cells (22, 45). A HDCs subtypes (Table 1) containing mixed features of the myeloid and lymphoid subtypes (54–58), and a subset called natural killer DCs (59) have been recently characterized. Finally, the DCs expressing CX3CR1 contribute to sustained inflammation in mice with diet-induced metabolic steatohepatitis (60). Additionally, CD40 expressing CD11c+ cells play a crucial role in protection against obesity-induced ectopic lipid storage and metabolic dysfunction, most likely via induction of Treg, however, during metabolic steatohepatitis, CD40 on CD11c+ cells contributes to liver inflammation (61).

**Table 1:** Dendritic cells subtypes (54–58).

**Dendritic cell subtype**	**Surface markers**	**Functions**	**Role in liver tolerance or disease**
*Myeloid Dendritic Cells* *Type 1 Dendritic Cells* 5% Blood 30% Liver	CD11c+ CD8α-CD11b+SIRPα+ CD103+ CD11b- BDCA3+ XCR1+ CLEC9A+	Depuration of apoptotic detritus Downregulation of TLR by DC Inhibition of CD8+ expansion Their depletion induce Th1 and Th17 Induce Treg survival by the expression of r expression of CD70 Induction of regulators as A20 modulating NF-κB signaling Apoptosis of T effector Cells by depleting tryptophan Negative incitement via CTLA4-CD80/CD86 or PD-1-PD-L1/ PD-L2 Secrete TGFB for FoxP3+ T reg cell generation Decrease in IFN-γ gene transcription	Reduction of liver inflammation and fibrosis Mediate tolerance by inhibition of NF-κB signaling Induce the development of T cell hyporesponsiveness
*Myeloid Dendritic Cells* *Type 2 Dendritic Cells* 95% Blood 70% Liver	CD11c+ CD103- CD11b+ BDCA1+ CD14+ SIRPα+	Propitiate an inflammatory setting Activation of T cells including MHCII Increase expression of CD40, CD80/CD86 Increase secretion of proinflammatory cytokines and chemokines Recruitment of macrophages into the liver	Increment of hepatic inflammation Inducing CD4+ Tcell-mediated immunity
*Plasmocytoid Dendritic Cells*	CD11cintCD45RAint CD11c+ SiglecH+ CD11b- CD103- BDCA2+ CD14+ CD123+	Developed in periphery utilize CCR9/α4 integrin signals In steady state, contribute to the maintenance of tolerance In steady state express low levels of MHCII contributing to T cell disregard In active state upregulate MHCII molecules inducing T cell proliferation Produce IFN-1 and IL10contributing to T reg formation, and IDO and PDL1 increasing Treg density Respond to viral infections secreting IFN1	Represent the most important cell type in antiviral innate immunity Reduced number in Liver cirrhosis Defense against viral infection by cross-talk with NK cells Produce type I interferons (IFN-alpha/beta) in response to toll like receptor

In human, The CD11C+ cDC2 may have an important role in fibrosis development in obesity induced metabolic steatohepatitis patients (62). A transcriptional and immune profiling of patients with metabolic steatohepatitis was recently conducted (63) showing that cDC2 were positively correlated with metabolic steatohepatitis progression while cDC1 and pDC were associated with a negative hepatic expression of genes involved in immune regulation and antigenic presentation. Nonetheless, the actual role of DCs in the pathogenesis of metabolic steatohepatitis is still a matter of debate, shown contrasting results depending on the experimental setting (24, 27). It is unclear whether DCregs constitute an independent DC subset or represent a specific functional state of DCs. In fact, most DC subsets can exert regulatory function through T cell anergy, T cell deletion, and Treg induction (64, 65). Furthermore, nomenclature differentiates HDCs based on lipid content with high-lipid liver DCs inducing robust T-cell activation and cytokine secretion whereas low-lipid DC promote immune tolerance (26).

#### Dendritic Cells Role in Hepatic Inflammation and Hepatocytes Cell Death

The DCs exist in mature or immature conditions, these latter is the most prevalent in peripheral tissues. *In vitro*, immature dendritic cells capture antigens, phagocyte particles (66), form pinocytic vesicles, and express receptors to mediate endocytosis (C-type lectin receptors, DEC-205, Fcγ and Fcε receptors) (67). The IDCs may prompt tolerance by deletion of T cells and induction of regulatory T cells, and other diverse mechanisms. The IDCs express low levels of surface MHC class I and II and costimulatory molecules (CD86 and CD40). They capture self-antigens and innocuous environmental proteins and target them to MHC II in the lysosomes, but they are not used for the formation of MHC II-peptide complexes (68). The DCs play a role in developing immune tolerance by presenting self-antigens to T cells, deleting autoreactive lymphocytes, and inducing regulatory T-cell formation, which induces tolerance by suppressing the responses of T cells to stimuli (69).

**Figure 2:**
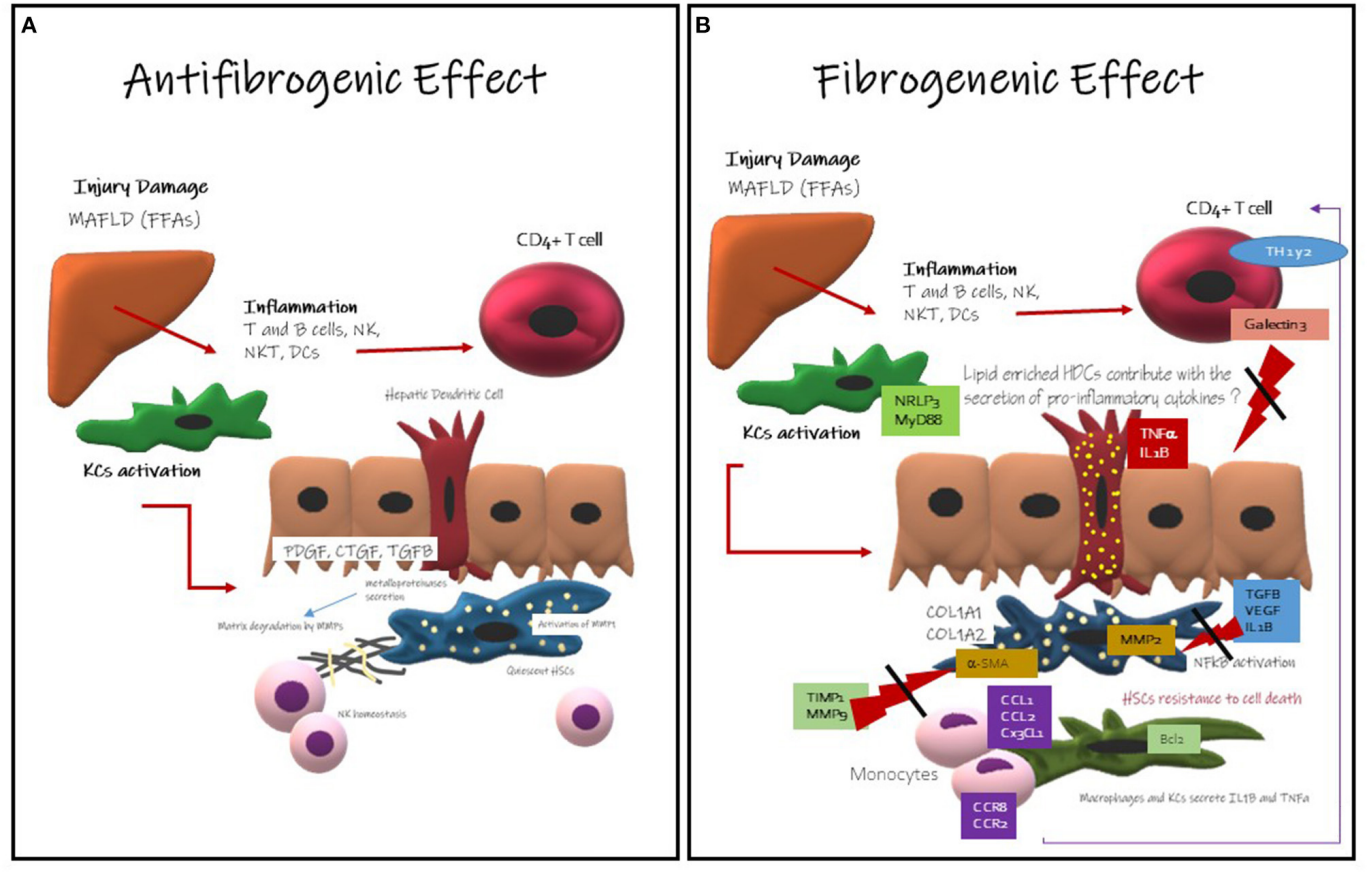
Hepatic Dendritic Cells Role in development and progression of liver fibrosis. **(A)** Antifibrogenic effects of Hepatic Dendritic Cells: they might contribute to the regression of liver fibrosis activating metalloproteinase like MMP-dependent mechanism, it is well-known that HDCs secrete metalloproteinase. Metalloproteinase are involved in the degeneration of the extracellular matrix. MMP-9 is involved in fibrosis regression and ECM remodeling. They also promote NK cells homeostasis that are mainly antifibrogenic. **(B)** Fibrogenic Effects of Hepatic Dendritic Cells: this effect is less understood, but here has been described that HDCs CD11c-positive induce NF-κB activation in HSCs via TNF and IL-1 production promoting HSCs survival. HDCs might have an enhanced antigen presentation activity and cytokine production and probably TLR stimulation. HDCs might contribute with the inflammatory microenvironment The immune cells more likely to promote fibrosis are KCs (22).

When DCs are exposed to immune or inflammatory signals (such as microbial products and proinflammatory cytokines), they undergo maturation (70) and they are directed to the T-cell areas of the lymphoid organs from the peripheral tissues (71). The DCs bring antigens to T cells and express costimulatory molecules, facilitating the induction of the immune response. Initially, fragments of antigens bounded to major histocompatibility complex (MHC) molecules are recognized by receptors on the T cells. Specifically, MHC class I molecules stimulate cytotoxic T cells (CD8+), and MHC II molecules stimulate T helper cells (CD4+) (72). Self-antigens captured and presented by DCs may induce and maintain tolerance (73), in addition to induction of immunity previously discussed (74). Mature dendritic cells (MDCs) have a reduced capacity to take up antigens but they can better stimulate T cells by the processed and presented antigens (67). Maturation causes an increase in the production and redistribution from the intracellular compartments to the plasma membrane of MHC II-peptide complexes (68, 75), expression of costimulatory molecules to increase adhesion and signaling in the T cells, and the production of cytokines in the HDCs (76). The adaptive immune response will end up orchestrating the chronicity of inflammation and liver damage in metabolic steatohepatitis patients (Figure 2). Differences have been observed between animal models and human dendritic cells, which may be important in the research field (Table 2).

**Table 2:** Dendritic Cells knowledge in humans and mice models (22, 52, 53, 77–84).

**Knowledge**	**Humans**	**Animal models (mice)**
*Subsets and surface markers*	DCs are lineage HLA-DR+ DC-SIGN (DC-specific intercellular adhesion molecule-3 [ICAM-3] grabbing non-integrin): immature DCs. **High and low lipid content DCs** **Myeloid DC** (CD11c+, CD11b+ lineage (lin) – BDCA-1+ (90% of human mDCs) or CD11c+CD123– lin– HLA-DR+ **CD8α+DC** not detected **Plasmocytoid DC** (BDCA-2+, BDCA-4+, or lin– HLA-DR+ CD11c– CD123^hi^ or CD4+CD11c–) **Natural Killer DC** not detected	**Lymphoid** (CD8α+, B220–, CD11b–) **Myeloid** (CD8α-, B220–, CD11b+), **Plasmacytoid** (CD11cloB220+Ly−6C+CD11b– or PDCA-1+) **Mixed lymphoid** **+** **myeloid** (CD8α –, B220–, CD11b–, DX5–) **High and low lipid content DCs** **CD8α+DC** (CD11c+CD8α+ CD11b-) **Natural Killer DC** (CD11c+NK1.1+)
*Location*	Perivenular region, portal space, few in the parenchyma.	
*Role in inflammation or tolerance*	Activate T cell responses Activate immune Th1 or Th2. Increases inflammation via DAMPS (HMGB1) and LPS by TLR4 activation. Endotoxin tolerance via an alteration of TLR responses Maturation is triggered by TNF receptor or TLR1-10 Interaction of DC MHCII and Cd4+Tcell cause their differentiation from Th1 to Th2. Induce IL17 to produce Th17 and Treg Hepatic CD141(+) DCs showed pro-inflammatory function in lymphocyte reactions, causing the production of IFNγ and IL17 by T cell. CD141(+) DCs were significantly depleted in liver diseases. pDc High responsiveness to TLR 7/8/9, secrete IFNα in hepatotropic viral infections Myeloid DCs Produce high levels of CXCL10, IL12p70, IFNβ, IFNλ after TLR stimulation Depleting CD11c+ DCs or CD103+ DCs reduced proinflammatory cytokine and chemokine expression	Activate T helper cells Maturation is triggered by TNF receptor or TLR1-13 HDCs CD103+= regulates immunogenic response to hepatotropic viral infection supporting CD8+ T cell response CX3CR1+ moDCs in inflammatory setting cause the development of HDCs with pro-inflammatory and immune-stimulating activities. Might turn to Th1 or Th2 depending on the secreted cytokines (IFNγ or IL4) CD39 expression might protect against inflammation by hyporesponsiveness to TLR4 Interaction with NK cells (NKG2A receptor) PDL1^hi^ DCs play a role in regulation of alloimmunity and tolerance CX3CR1blocker CX3AT ameliorates hepatic inflammation CD103+ cDC1 protective DC subtype that influences the pro-anti-inflammatory balance
	Secrete IL10 reducing the responsiveness to T cell and promoting Treg generation.	
	Induce Treg differentiation by PD-L1 expression	
	DCs influence the hepatic cytokine microenvironment	
	Maturation of naïve T cells into Foxp3+Treg	
*Role in Liver Fibrosis*	Role in inducing NK promoting CD8+ T cell differentiation.	Expansion of DCs might ameliorate liver fibrosis regression
	Secrete metalloproteinase	
	Regression of fibrosis via MMP-9 mechanism	
	DCs possible portrayal in fibrosis by controlling other immune cells.	

These opposing roles of HDCs are likely contributing to the conflicting results reported for their role in liver injury.

In healthy livers, HDC display a predominant immature phenotype, characterized by a low capacity to endocytose antigens and to stimulate T-lymphocytes accompanied with a high production of kynurenine (85), IL-10 and IL-27 promoting the differentiation of CD4+ T cells into regulatory T cells (Treg) (86). Immature HDCs are resilient to maturation stimulus like gut LPS and gut bacterial products (50). While, in the inflammatory state, they maturate and facilitate the overthrow of monocytes, the production of proinflammatory cytokines and chemokines. In this inflammatory environment, they develop an important capacity to respond to TLR, to activate NKT cells and to promote T-cell proliferation (77). They also expand and activate, changing to a pro-inflammatory immunogenic phenotype as efficient APC and a source of pro-inflammatory cytokines like TNF-α and IL6, provoking oxidative stress and the activation of stellate cells (22, 25, 45, 50).

Henning et al. (32) demonstrated that HDC *in* MAFLD mice models exhibit maturation and increase the production of pro-inflammatory cytokines and chemokines. In injured liver, enhancing the differentiation of monocyte derived dendritic cells is responsible for HDCs proinflammatory responses. While, ablation of HDC might be deleterious through triggering an increased hepatic damage and aggravating sterile inflammation and apoptosis. Subsequently, the activation of pro-apoptotic mediators such as p53, Fas ligand and BCl2 may lead to acceleration of fibrosis. Sutti et al. (51) have shown also in a mice model that HDCs promotes mainly hepatic inflammation by the upregulation of fractalkine (CX3CL1) linked to the expansion of CX3CR1+moDCs. The authors demonstrate in experimental settings that CX^3^CR1^high^ myeloid HDCs provoke the perpetuation of the inflammation and injury of the liver, remarking that CX^3^CR1 signals mediate the differentiation of myeloid HDCs; they also showed that the blockage of CX3CR1 ameliorates hepatic inflammation and damage. While, Heier et al. (53) identified a specific murine dendritic cell as a protective DC subtype that influences the pro–anti-inflammatory balance and protects the liver from metabolic damage, myeloid cells including HDCs influence the complex network responsible for sustaining lobular inflammation in metabolic steatohepatitis. In experimental animal models, myeloid HDCs (DC1) identified with the CD103+ marker appear to have a protective role in the liver by founding that the transference of CD103+ cDC1 to a Batf3-/- deficient murine cohort reduced inflammatory monocyte recruitment, liver CCL2 expression and serum transaminases without affecting the extent of steatosis, and the selective depletion of this subgroup resulted in initiation, worsening or acceleration of the inflammation or fibrosis (53), whereas the depletion of a proinflammatory subgroup may result in a beneficial effect on the disease (22, 28, 30, 69). Interestingly, the immune-stimulating and pro-inflammatory phenotype of HDCs seems to be associated with a high-lipid content in the cell (47). In conclusion, each one of the specific subgroups of HDCs have different roles in the balance of hepatic fibro inflammatory process, now we know the value of the accurate identification for avoiding methodological mistakes during research.

Collectively, these data suggest that HDC may have a dual roles implicated in both the progression and resolution of metabolic steatohepatitis. Future research would be required to identify, specific DCs subsets of HDCs that might have a protective role in hepatic inflammation and those that enhance inflammatory responses to pave the way for HDC based therapeutic targets for MAFLD.

#### The Role of Dendritic Cells in Hepatic Lipid Accumulation

HDCs are implicated in adipogenesis and lipid metabolism lipid synthesis and hepatic lipid accumulation through the activation of acetylCoA carboxylase (50).

It has been shown that dendritic cells compile lipids, lipid enriched DCs have a powerful pro-inflammatory activity compared to lipid-low DCs that are more tolerogenic. It has been demonstrated by Ibrahim et al. (26) that high lipid DCs importantly express MHC II with an efficient T cell proliferation and activation inducing the secretion of proinflammatory cytokines such as (IL-6, TNF-α, IL-2, beneath others); enhancing their immune stimulatory capacity. They also showed that high DC notably activate NK and NKT cells as well as a TN-α dependent adipogenesis and ER stress.

Recently Aarts et al. (61) showed that in a mice metabolic steatohepatitis model that CD40CD11 cells have higher hepatic and plasmatic cholesterol levels. The absence of CD40^+^ T cells on CD11c cells decrease liver inflammation and protect against metabolic syndrome by inducing T-reg cells.

## Clinical Implications of Reticuloendothelial System Cells in Lipotoxicity in Metabolic Steatohepatitis

HDCs play a key role in the tolerance but also in the development of metabolic steatohepatitis. Lipotoxicity is portrayed by the increased concentration of toxic lipid and it is known that it plays an important role in the pathogenesis of MAFLD. The excessive lipid concentration within the liver enhances the maturation of HDcs and the set up lipid enriched HDCs, which regulates immunogenic responses (26). The accumulation of cholesterol leads to macrophages and KCs activation, with consequent inflammation (25). Also this excessive quantity of toxic lipids in the liver causes endoplasmic reticulum (ER) and mitochondrial stress, by creating an aberrant response to misfolded proteins in the ER (87, 88), and ROS formation (87) with parallel alteration on β-oxidation process due to the overwhelmed capacity of the mitochondria to metabolize the excessive free fatty acids (FFAs) (89). ER stress likewise promotes proteotoxicity and proapoptotic signals through a rapid decay of selected microRNAs that would normally suppress apoptosis (90–93). There are several pathways related to hepatocytes apoptosis among them the C/EBP homologous protein (CHOP) a proapoptotic signal activated by PERK and ATF6 pathways (90), the c-Jun N-terminal kinase (JNK) (92), the Bcl-2-associated X protein (BAX) and Bcl-2 homologous antagonist killer (BAK) pathways (94). During mitochondrial dysfunction, ROS cause a decreased levels of adenosine triphosphate (ATP) and depletion or inhibition of antioxidant molecules, mainly thioredoxin and glutathione (95, 96). Finally, the oxidative stress caused by the lipid peroxidation breakdown products, like malondialdehyde (MDA), malondialdehyde-acetaldehyde (MAA), and 4- hydroxynonenal, as well as phosphocholine (PC)-containing oxidized phospholipids formally called oxidation-specific epitopes (OSEs) (97) perpetuating protein oxidation and lipid peroxidation and triggering adaptive immune response through a group of protein adducts (49). Damaged mitochondria inflict the subsequent necrosis of hepatocytes releasing mitochondria-derived DAMPs (such as the high mobility group box protein (HMGB1), nucleic acids, histones, ATP and uric acid) which can be recognized by the HDCs and induce an innate immunogenic response (98–100). This HMGB1 activates several TLR, perpetuating inflammatory damage, recruiting neutrophils and causing the amplification of the damage (25). It has also been described, that the mitochondrial DNA (mtDNA) interacts with TLR-9 on KCs and HSCs promoting the innate immune and fibrogenic responses (98–101). Finally, the release of extracellular vesicles (EVs) from hepatocytes stimulate the activation of HDCs. Recently has been discovered that exosomes, a subgroup of EVs, transport the chemokine CXCL10 and ceramides to KCs, which recruit neutrophils via IL-8 (25) and activate macrophages via sphingosine-1-phosphate (24). Further, palmitate induce the liberation of EVs by the hepatocytes containing TNF-related apoptosis-inducing ligand (TRAIL), an important proapoptotic protein with the ability to activate macrophages (102). The inflammatory mediators and cytokines, endotoxins, adipokines, chemokines (103) and other cell damage signals released by lipotoxic hepatocytes promote sterile inflammation with activation of innate (such as KCs, HDCs, neutrophils, etc.) and adaptive immune cells (such as CD4+ and CD8+ T cells, B cells, T regulator cells, NKT cells, etc.). The different stimulus during lipotoxic and inflammatory cell damage in addition to metabolic derangements in the HSCs stimulate their differentiation of into myofibroblasts, which is the most important mechanisms involved in further maintenance and progression of metabolic steatohepatitis into fibrosis and finally cirrhosis (22).

## Conclusions

Dendritic Cells plays a key role in the maintenance of hepatic immune homeostasis and tolerance contributing to the suppression of the expansion of CD8+ T cells, thereby to their deletion. They secrete anti-inflammatory cytokines maintaining the quiescence of the HSCs. They also promote the refractoriness of TLR4 to LPS in conjunction with the clearance of cellular debris. Even though, they are responsible of the antigen presentation to T cells, they interact with Kupffer cells enhancing this tolerance within the liver secreting IL-10 under reliable conditions. When inflammatory pathways are produce in the liver, HDCs react enhancing this liver inflammation. It is important to have a better understanding of these amazing cells to deeply discern metabolic steatohepatitis physiopathology.

## Author Contributions

NM-S, JC-G, BB-F, and ME contributed to the conceptualization and the writing of the manuscript. All authors contributed to the article and approved the submitted version.

## Conflict of Interest

The authors declare that the research was conducted in the absence of any commercial or financial relationships that could be construed as a potential conflict of interest.

## Abbreviations

MAFLD: Metabolic Associated Fatty Liver Disease
DC: Dendritic Cells
HDC: Hepatic Dendritic Cells
KCs: Kupffer Cells
LSEC: Liver Sinusoidal Endothelial Cells
DAMPS: Damage associated Molecular patterns
TLR: Toll like Receptor
LPS: Lypopolysaccharide
PDL1: Programmed cell Death Ligand 1
Treg: regulatory T cells.
